# 1-(3,5-Dimeth­oxy­phen­yl)-2-(4-fluoro­phen­yl)-4,5-dimethyl-1*H*-imidazole

**DOI:** 10.1107/S1600536811055012

**Published:** 2012-01-07

**Authors:** S. Rosepriya, A. Thiruvalluvar, K. Saravanan, J. Jayabharathi, Ray J. Butcher

**Affiliations:** a PG Research Department of Physics, Rajah Serfoji Government College (Autonomous), Thanjavur 613 005, Tamilnadu, India; b Department of Chemistry, Annamalai University, Annamalai Nagar 608 002, Tamilnadu, India; c Department of Chemistry, Howard University, 525 College Street NW, Washington, DC 20059, USA

## Abstract

In the title compound, C_19_H_19_FN_2_O_2_, the imidazole ring is essentially planar [maximum deviation = 0.0030 (8) Å] and makes dihedral angles of 66.45 (7) and 29.98 (7)° with the benzene rings attached to the ring N and C atoms, respectively. The dihedral angle between the two benzene rings is 64.79 (7)°. A C—H⋯π inter­action is found in the crystal structure. The two meth­oxy groups were found to be disordered over two sets of sites with occupancy factors of 0.803 (4) and 0.197 (4). The F atom is disordered over two sites with occupancy factors of 0.929 (4) and 0.071 (4).

## Related literature

For general background to the use of imidazole derivatives as drugs, see: Dooley *et al.* (1992 ▶); Jackson *et al.* (2000 ▶); Banfi *et al.* (2006 ▶). For a related structure and applications of imidazole derivatives, see: Rosepriya *et al.* (2011 ▶).
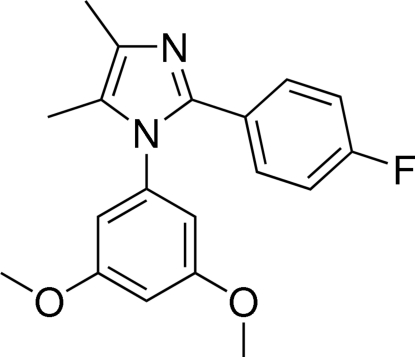


## Experimental

### 

#### Crystal data

C_19_H_19_FN_2_O_2_
*M* *_r_* = 326.36
Monoclinic, 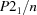

*a* = 6.9654 (1) Å
*b* = 17.8520 (3) Å
*c* = 13.7121 (3) Å
β = 97.833 (2)°
*V* = 1689.14 (5) Å^3^
*Z* = 4
Cu *K*α radiation
μ = 0.75 mm^−1^
*T* = 295 K
0.47 × 0.38 × 0.16 mm

#### Data collection

Oxford Diffraction Xcalibur Ruby Gemini diffractometer
Absorption correction: multi-scan *CrysAlis PRO* (Oxford Diffraction, 2010 ▶) *T*_min_ = 0.558, *T*_max_ = 1.000
7744 measured reflections
3533 independent reflections
2723 reflections with *I* > 2σ(*I*)
*R*_int_ = 0.024

#### Refinement

*R*[*F* ^2^ > 2σ(*F* ^2^)] = 0.046
*wR*(*F* ^2^) = 0.138
*S* = 1.06
3533 reflections
240 parameters
H-atom parameters constrained
Δρ_max_ = 0.21 e Å^−3^
Δρ_min_ = −0.18 e Å^−3^

### 

Data collection: *CrysAlis PRO* (Oxford Diffraction, 2010 ▶); cell refinement: *CrysAlis PRO*; data reduction: *CrysAlis PRO*; program(s) used to solve structure: *SHELXS97* (Sheldrick, 2008 ▶); program(s) used to refine structure: *SHELXL97* (Sheldrick, 2008 ▶); molecular graphics: *ORTEP-3* (Farrugia, 1997 ▶); software used to prepare material for publication: *PLATON* (Spek, 2009 ▶).

## Supplementary Material

Crystal structure: contains datablock(s) global, I. DOI: 10.1107/S1600536811055012/hg5156sup1.cif

Structure factors: contains datablock(s) I. DOI: 10.1107/S1600536811055012/hg5156Isup2.hkl

Supplementary material file. DOI: 10.1107/S1600536811055012/hg5156Isup3.cdx

Supplementary material file. DOI: 10.1107/S1600536811055012/hg5156Isup4.cml

Additional supplementary materials:  crystallographic information; 3D view; checkCIF report

## Figures and Tables

**Table 1:** Hydrogen-bond geometry (Å, °) *Cg*1 is the centroid of the N1/C2/N3/C4/C5 ring.

*D*—H⋯*A*	*D*—H	H⋯*A*	*D*⋯*A*	*D*—H⋯*A*
C23—H23⋯*Cg*1^i^	0.93	2.99	3.8714 (16)	159

Symmetry code: (i) 
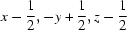
.

**Fig. 1.**
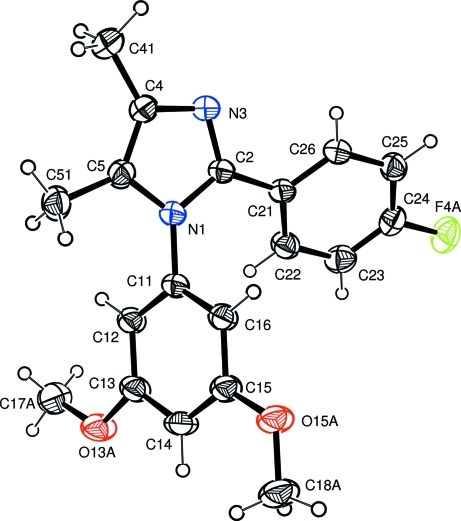
The molecular structure of the title compound, with displacement ellipsoids drawn at the 30% probability level. H atoms are shown as small spheres of arbitrary radius. The minor component of methoxy and F atoms are omitted for clarity.

## supplementary crystallographic information

### Comment

For multidrug-resistant Tuberculosis (Dooley *et al.*,(1992)), antifungal and antimycobacterial activity (Banfi *et al.* 2006), and bactericidal effects (Jackson *et al.* 2000), the use of imidazole based compounds were reported. Rosepriya *et al.* 2011 have reported the crystal structure of 1-(3,5-Dimethylphenyl)-2-(4-fluorophenyl)-4,5-dimethyl-1*H*-imidazole. As part of our research (Rosepriya *et al.* 2011), we have synthesized the title compound (I) and report its crystal structure here.

In the title compound, Fig. 1, C_19_H_19_FN_2_O_2_, the imidazole ring is essentially planar [maximum deviation of 0.0030 (8) Å for C4]. The imidazole ring makes dihedral angles of 66.45 (7) and 29.98 (7)° with the benzene rings attached to nitrogen and carbon, respectively. The dihedral angle between the two benzene rings is 64.79 (7)°. A C23—H23···π interaction involving the imidazole (N1,C2,N3,C4,C5) ring is found in the crystal structure (Table 1). The two methoxy groups at C13 and C15 were found to be disordered over two positions with occupancy factors of 0.803 (4) and 0.197 (4). The F atom at C24 is disordered over two positions with occupancy factors of 0.929 (4) and 0.071 (4).

### Experimental

To pure butane-2,3-dione (1.48 g, 15 mmol) in ethanol (10 ml), 3,5-dimethoxyaniline (2.29 g, 15 mmol), ammonium acetate (1.15 g, 15 mmol) and 4-fluorobenzaldehyde (1.8 g, 15 mmol) was added over 1 hr with the temperature maintained at 333 K. The reaction mixture was refluxed for 7 days and extracted with dichloromethane. The solid separated was purified by column chromatography using Hexane: Ethyl acetate as the eluent. Yield: 2.20 g (45%). Crystals suitable for X-ray diffraction studies were grown by slow solvent evaporation of a solution of the compound in dichloromethane.

### Refinement

The H atoms were positioned geometrically and allowed to ride on their parent atoms, with C—H = 0.93 - 0.96 Å; *U*_iso_(H) = k*U*_eq_(C), where k = 1.5 for methyl and 1.2 for all other H atoms. The two methoxy groups were found to be disordered over two positions with occupancy factors of 0.803 (4) and 0.197 (4). The F atom is disordered over two positions with occupancy factors of 0.929 (4) and 0.071 (4).

### Crystal data

**Table:**

C_19_H_19_FN_2_O_2_	*F*(000) = 688
*M**_r_* = 326.36	*D*_x_ = 1.283 Mg m^−^^3^
Monoclinic, *P*2_1_/*n*	Melting point: 420 K
Hall symbol: -P 2yn	Cu *K*α radiation, λ = 1.54184 Å
*a* = 6.9654 (1) Å	Cell parameters from 3957 reflections
*b* = 17.8520 (3) Å	θ = 5.0–77.4°
*c* = 13.7121 (3) Å	µ = 0.75 mm^−^^1^
β = 97.833 (2)°	*T* = 295 K
*V* = 1689.14 (5) Å^3^	Plate, colourless
*Z* = 4	0.47 × 0.38 × 0.16 mm

### Data collection

**Table:**

Oxford Diffraction Xcalibur Ruby Gemini diffractometer	3533 independent reflections
Radiation source: Enhance (Mo) X-ray Source	2723 reflections with *I* > 2σ(*I*)
graphite	*R*_int_ = 0.024
Detector resolution: 10.5081 pixels mm^-1^	θ_max_ = 77.6°, θ_min_ = 5.0°
ω scans	*h* = −4→8
Absorption correction: multi-scan CrysAlis PRO (Oxford Diffraction, 2010)	*k* = −22→21
*T*_min_ = 0.558, *T*_max_ = 1.000	*l* = −17→17
7744 measured reflections	

### Refinement

**Table:**

Refinement on *F*^2^	Primary atom site location: structure-invariant direct methods
Least-squares matrix: full	Secondary atom site location: difference Fourier map
*R*[*F*^2^ > 2σ(*F*^2^)] = 0.046	Hydrogen site location: inferred from neighbouring sites
*wR*(*F*^2^) = 0.138	H-atom parameters constrained
*S* = 1.06	*w* = 1/[σ^2^(*F*_o_^2^) + (0.0917*P*)^2^ + 0.0185*P*] where *P* = (*F*_o_^2^ + 2*F*_c_^2^)/3
3533 reflections	(Δ/σ)_max_ = 0.001
240 parameters	Δρ_max_ = 0.21 e Å^−^^3^
0 restraints	Δρ_min_ = −0.18 e Å^−^^3^

### Special details

**Table:**

Geometry. Bond distances, angles *etc*. have been calculated using the rounded fractional coordinates. All su's are estimated from the variances of the (full) variance-covariance matrix. The cell e.s.d.'s are taken into account in the estimation of distances, angles and torsion angles
Refinement. Refinement of *F*^2^ against ALL reflections. The weighted *R*-factor *wR* and goodness of fit *S* are based on *F*^2^, conventional *R*-factors *R* are based on *F*, with *F* set to zero for negative *F*^2^. The threshold expression of *F*^2^ > 2σ(*F*^2^) is used only for calculating *R*-factors(gt) *etc*. and is not relevant to the choice of reflections for refinement. *R*-factors based on *F*^2^ are statistically about twice as large as those based on *F*, and *R*- factors based on ALL data will be even larger.A damping factor (DAMP 200 15 in the final refinement cycles) was applied to avoid large displacements of the less occupied methoxy and fluorine atoms with EADP F4A F4B, EADP O13A O13B, EADP O15A O15B, EADP C17A C17B, EADP C18A C18B.

### Fractional atomic coordinates and isotropic or equivalent isotropic displacement parameters (Å^2^)

**Table:**

	*x*	*y*	*z*	*U*_iso_*/*U*_eq_	Occ. (<1)
F4A	−0.29421 (17)	0.18280 (10)	0.14002 (10)	0.0827 (4)	0.929 (4)
O13A	0.7091 (3)	0.45839 (10)	0.22912 (15)	0.0748 (6)	0.803 (4)
O15A	0.2065 (3)	0.45966 (11)	0.43181 (19)	0.0728 (6)	0.803 (4)
N1	0.55071 (14)	0.23172 (5)	0.40039 (8)	0.0452 (3)	
N3	0.48548 (15)	0.11225 (6)	0.42587 (9)	0.0513 (3)	
C2	0.42077 (16)	0.17418 (6)	0.38065 (9)	0.0446 (3)	
C4	0.66338 (17)	0.12988 (7)	0.47766 (10)	0.0513 (3)	
C5	0.70736 (16)	0.20299 (7)	0.46281 (10)	0.0493 (3)	
C11	0.52171 (16)	0.30968 (6)	0.37642 (9)	0.0459 (3)	
C12	0.64059 (19)	0.34342 (7)	0.31551 (11)	0.0533 (4)	
C13	0.6101 (2)	0.41860 (8)	0.29299 (12)	0.0600 (4)	
C14	0.4653 (2)	0.45903 (7)	0.33020 (13)	0.0643 (5)	
C15	0.3491 (2)	0.42387 (7)	0.38983 (11)	0.0564 (4)	
C16	0.37798 (17)	0.34825 (7)	0.41430 (10)	0.0502 (4)	
C17A	0.8558 (4)	0.4227 (2)	0.1871 (3)	0.0874 (9)	0.803 (4)
C18A	0.1764 (5)	0.53712 (15)	0.4144 (3)	0.0865 (12)	0.803 (4)
C21	0.23453 (16)	0.17930 (6)	0.31576 (9)	0.0446 (3)	
C22	0.2032 (2)	0.22706 (8)	0.23517 (10)	0.0562 (4)	
C23	0.0262 (2)	0.22827 (9)	0.17545 (11)	0.0642 (4)	
C24	−0.11786 (19)	0.18160 (8)	0.19783 (11)	0.0571 (4)	
C25	−0.09381 (19)	0.13336 (7)	0.27609 (11)	0.0551 (4)	
C26	0.08443 (18)	0.13216 (7)	0.33465 (10)	0.0500 (3)	
C41	0.7816 (2)	0.07173 (10)	0.53699 (14)	0.0737 (5)	
C51	0.8802 (2)	0.24892 (9)	0.50026 (13)	0.0651 (5)	
C17B	0.898 (2)	0.4240 (10)	0.2150 (14)	0.0874 (9)	0.197 (4)
C18B	0.135 (2)	0.5285 (8)	0.3807 (13)	0.0865 (12)	0.197 (4)
O13B	0.7483 (14)	0.4621 (5)	0.2658 (7)	0.0748 (6)	0.197 (4)
O15B	0.1779 (16)	0.4481 (5)	0.4134 (10)	0.0728 (6)	0.197 (4)
F4B	−0.253 (3)	0.2120 (13)	0.1380 (16)	0.0827 (4)	0.071 (4)
H16	0.30133	0.32452	0.45545	0.0602*	
H17A	0.94989	0.40279	0.23819	0.1309*	0.803 (4)
H12	0.73740	0.31646	0.29069	0.0639*	
H14	0.44718	0.50951	0.31489	0.0771*	
H22	0.30229	0.25856	0.22119	0.0675*	
H23	0.00548	0.26006	0.12131	0.0770*	
H25	−0.19419	0.10232	0.28952	0.0662*	
H26	0.10444	0.09926	0.38765	0.0600*	
H41A	0.86731	0.04817	0.49709	0.1106*	
H41B	0.69719	0.03475	0.55904	0.1106*	
H41C	0.85625	0.09493	0.59293	0.1106*	
H51A	0.96450	0.22046	0.54767	0.0976*	
H51B	0.83938	0.29349	0.53081	0.0976*	
H51C	0.94812	0.26244	0.44646	0.0976*	
H17B	0.91718	0.45800	0.14857	0.1309*	0.803 (4)
H17C	0.80126	0.38258	0.14554	0.1309*	0.803 (4)
H18A	0.29026	0.56430	0.44132	0.1298*	0.803 (4)
H18B	0.06803	0.55363	0.44518	0.1298*	0.803 (4)
H18C	0.15032	0.54600	0.34477	0.1298*	0.803 (4)
H17D	0.99679	0.40337	0.26325	0.1309*	0.197 (4)
H17E	0.95528	0.45968	0.17517	0.1309*	0.197 (4)
H17F	0.83856	0.38452	0.17401	0.1309*	0.197 (4)
H18D	0.25069	0.55810	0.39539	0.1298*	0.197 (4)
H18E	0.03553	0.54860	0.41504	0.1298*	0.197 (4)
H18F	0.09270	0.52961	0.31114	0.1298*	0.197 (4)

### Atomic displacement parameters (Å^2^)

**Table:**

	*U*^11^	*U*^22^	*U*^33^	*U*^12^	*U*^13^	*U*^23^
F4A	0.0562 (6)	0.1013 (10)	0.0835 (6)	0.0048 (5)	−0.0161 (5)	−0.0034 (7)
O13A	0.0855 (9)	0.0547 (6)	0.0943 (13)	0.0053 (6)	0.0491 (9)	0.0201 (8)
O15A	0.0821 (9)	0.0435 (8)	0.1025 (12)	0.0134 (6)	0.0473 (8)	0.0021 (7)
N1	0.0446 (4)	0.0365 (4)	0.0546 (5)	−0.0007 (3)	0.0076 (4)	−0.0003 (4)
N3	0.0490 (5)	0.0410 (5)	0.0638 (6)	0.0016 (4)	0.0079 (4)	0.0075 (4)
C2	0.0466 (5)	0.0357 (5)	0.0523 (6)	−0.0006 (4)	0.0092 (4)	0.0016 (4)
C4	0.0472 (5)	0.0477 (6)	0.0594 (7)	0.0056 (5)	0.0090 (5)	0.0059 (5)
C5	0.0443 (5)	0.0474 (6)	0.0564 (6)	0.0030 (4)	0.0075 (4)	−0.0035 (5)
C11	0.0481 (5)	0.0349 (5)	0.0552 (6)	−0.0017 (4)	0.0086 (4)	−0.0018 (4)
C12	0.0532 (6)	0.0419 (6)	0.0685 (8)	0.0010 (5)	0.0218 (5)	−0.0016 (5)
C13	0.0636 (7)	0.0450 (6)	0.0767 (9)	−0.0020 (5)	0.0287 (6)	0.0070 (6)
C14	0.0749 (8)	0.0362 (6)	0.0872 (10)	0.0049 (5)	0.0309 (7)	0.0091 (6)
C15	0.0604 (7)	0.0421 (6)	0.0707 (8)	0.0061 (5)	0.0237 (6)	−0.0004 (5)
C16	0.0528 (6)	0.0414 (6)	0.0589 (7)	−0.0013 (5)	0.0168 (5)	0.0020 (5)
C17A	0.0810 (14)	0.0824 (12)	0.110 (2)	0.0107 (12)	0.0532 (13)	0.0286 (14)
C18A	0.0976 (16)	0.0484 (10)	0.123 (3)	0.0222 (10)	0.0493 (15)	0.0015 (12)
C21	0.0472 (5)	0.0360 (5)	0.0510 (6)	0.0010 (4)	0.0080 (4)	−0.0026 (4)
C22	0.0589 (7)	0.0536 (7)	0.0559 (7)	−0.0060 (5)	0.0070 (5)	0.0071 (6)
C23	0.0724 (8)	0.0631 (8)	0.0545 (7)	0.0051 (6)	−0.0003 (6)	0.0091 (6)
C24	0.0488 (6)	0.0609 (8)	0.0592 (7)	0.0081 (5)	−0.0011 (5)	−0.0104 (6)
C25	0.0484 (6)	0.0497 (6)	0.0677 (8)	−0.0033 (5)	0.0094 (5)	−0.0097 (6)
C26	0.0531 (6)	0.0394 (5)	0.0575 (7)	−0.0017 (5)	0.0077 (5)	0.0014 (5)
C41	0.0584 (7)	0.0687 (9)	0.0917 (11)	0.0116 (7)	0.0019 (7)	0.0249 (8)
C51	0.0499 (6)	0.0632 (8)	0.0799 (10)	−0.0034 (6)	0.0008 (6)	−0.0123 (7)
C17B	0.0810 (14)	0.0824 (12)	0.110 (2)	0.0107 (12)	0.0532 (13)	0.0286 (14)
C18B	0.0976 (16)	0.0484 (10)	0.123 (3)	0.0222 (10)	0.0493 (15)	0.0015 (12)
O13B	0.0855 (9)	0.0547 (6)	0.0943 (13)	0.0053 (6)	0.0491 (9)	0.0201 (8)
O15B	0.0821 (9)	0.0435 (8)	0.1025 (12)	0.0134 (6)	0.0473 (8)	0.0021 (7)
F4B	0.0562 (6)	0.1013 (10)	0.0835 (6)	0.0048 (5)	−0.0161 (5)	−0.0034 (7)

### Geometric parameters (Å, °)

**Table:**

F4A—C24	1.3683 (19)	C23—C24	1.371 (2)
F4B—C24	1.28 (2)	C24—C25	1.368 (2)
O13A—C17A	1.394 (4)	C25—C26	1.3838 (19)
O13A—C13	1.383 (3)	C12—H12	0.9300
O13B—C17B	1.494 (19)	C14—H14	0.9300
O13B—C13	1.329 (10)	C16—H16	0.9300
O15A—C15	1.372 (3)	C17A—H17A	0.9600
O15A—C18A	1.414 (3)	C17A—H17B	0.9600
O15B—C15	1.349 (11)	C17A—H17C	0.9600
O15B—C18B	1.521 (17)	C17B—H17F	0.9600
N1—C2	1.3714 (14)	C17B—H17E	0.9600
N1—C5	1.3898 (16)	C17B—H17D	0.9600
N1—C11	1.4379 (14)	C18A—H18C	0.9600
N3—C4	1.3780 (17)	C18A—H18B	0.9600
N3—C2	1.3167 (16)	C18A—H18A	0.9600
C2—C21	1.4725 (16)	C18B—H18F	0.9600
C4—C5	1.3623 (18)	C18B—H18E	0.9600
C4—C41	1.495 (2)	C18B—H18D	0.9600
C5—C51	1.4889 (19)	C22—H22	0.9300
C11—C16	1.3740 (17)	C23—H23	0.9300
C11—C12	1.3909 (18)	C25—H25	0.9300
C12—C13	1.3870 (19)	C26—H26	0.9300
C13—C14	1.392 (2)	C41—H41B	0.9600
C14—C15	1.378 (2)	C41—H41A	0.9600
C15—C16	1.3990 (18)	C41—H41C	0.9600
C21—C22	1.3892 (18)	C51—H51C	0.9600
C21—C26	1.3936 (17)	C51—H51A	0.9600
C22—C23	1.384 (2)	C51—H51B	0.9600
			
C13—O13A—C17A	119.0 (2)	C15—C14—H14	120.00
C13—O13B—C17B	116.3 (9)	C11—C16—H16	121.00
C15—O15A—C18A	118.9 (2)	C15—C16—H16	121.00
C15—O15B—C18B	112.1 (9)	O13A—C17A—H17A	109.00
C5—N1—C11	124.89 (10)	O13A—C17A—H17B	110.00
C2—N1—C5	106.66 (9)	O13A—C17A—H17C	109.00
C2—N1—C11	127.70 (10)	H17A—C17A—H17B	110.00
C2—N3—C4	106.08 (10)	H17A—C17A—H17C	109.00
N1—C2—C21	125.18 (10)	H17B—C17A—H17C	109.00
N3—C2—C21	123.61 (10)	H17E—C17B—H17F	109.00
N1—C2—N3	111.20 (10)	H17D—C17B—H17F	109.00
C5—C4—C41	128.92 (12)	O13B—C17B—H17E	109.00
N3—C4—C5	110.28 (11)	O13B—C17B—H17F	110.00
N3—C4—C41	120.78 (12)	O13B—C17B—H17D	109.00
C4—C5—C51	131.59 (12)	H17D—C17B—H17E	109.00
N1—C5—C51	122.63 (11)	O15A—C18A—H18C	109.00
N1—C5—C4	105.78 (10)	H18A—C18A—H18B	109.00
N1—C11—C16	119.05 (10)	O15A—C18A—H18B	109.00
C12—C11—C16	122.28 (11)	O15A—C18A—H18A	109.00
N1—C11—C12	118.67 (10)	H18A—C18A—H18C	109.00
C11—C12—C13	117.85 (12)	H18B—C18A—H18C	109.00
O13A—C13—C14	114.49 (14)	O15B—C18B—H18D	109.00
O13A—C13—C12	124.31 (14)	H18E—C18B—H18F	110.00
O13B—C13—C14	112.9 (4)	H18D—C18B—H18F	109.00
C12—C13—C14	121.10 (13)	O15B—C18B—H18E	110.00
O13B—C13—C12	122.2 (4)	O15B—C18B—H18F	110.00
C13—C14—C15	119.66 (12)	H18D—C18B—H18E	109.00
C14—C15—C16	120.34 (12)	C21—C22—H22	120.00
O15A—C15—C14	123.67 (14)	C23—C22—H22	120.00
O15A—C15—C16	115.94 (14)	C24—C23—H23	121.00
O15B—C15—C16	110.8 (4)	C22—C23—H23	121.00
O15B—C15—C14	127.3 (5)	C24—C25—H25	121.00
C11—C16—C15	118.77 (12)	C26—C25—H25	121.00
C2—C21—C26	118.14 (11)	C21—C26—H26	119.00
C2—C21—C22	123.28 (11)	C25—C26—H26	119.00
C22—C21—C26	118.55 (11)	C4—C41—H41B	109.00
C21—C22—C23	120.72 (13)	C4—C41—H41C	109.00
C22—C23—C24	118.57 (14)	H41A—C41—H41B	109.00
C23—C24—C25	122.89 (13)	C4—C41—H41A	109.00
F4B—C24—C23	95.5 (10)	H41A—C41—H41C	109.00
F4B—C24—C25	140.3 (10)	H41B—C41—H41C	109.00
F4A—C24—C25	117.94 (13)	H51B—C51—H51C	109.00
F4A—C24—C23	119.18 (14)	H51A—C51—H51B	109.00
C24—C25—C26	117.98 (12)	H51A—C51—H51C	109.00
C21—C26—C25	121.28 (12)	C5—C51—H51A	109.00
C11—C12—H12	121.00	C5—C51—H51B	109.00
C13—C12—H12	121.00	C5—C51—H51C	109.00
C13—C14—H14	120.00		
			
C17A—O13A—C13—C14	−179.4 (2)	C41—C4—C5—N1	178.65 (14)
C17A—O13A—C13—C12	−3.0 (3)	N3—C4—C5—C51	−179.23 (14)
C18A—O15A—C15—C14	−0.6 (3)	C41—C4—C5—C51	−1.1 (3)
C18A—O15A—C15—C16	176.7 (2)	C16—C11—C12—C13	−0.2 (2)
C2—N1—C5—C4	−0.21 (14)	N1—C11—C16—C15	−179.31 (12)
C11—N1—C2—N3	−170.50 (11)	C12—C11—C16—C15	0.7 (2)
C11—N1—C2—C21	10.84 (19)	N1—C11—C12—C13	179.83 (12)
C11—N1—C5—C51	−9.77 (19)	C11—C12—C13—C14	0.1 (2)
C5—N1—C2—C21	−178.82 (11)	C11—C12—C13—O13A	−176.12 (16)
C2—N1—C11—C16	60.35 (17)	O13A—C13—C14—C15	176.05 (16)
C5—N1—C11—C12	71.66 (16)	C12—C13—C14—C15	−0.5 (2)
C5—N1—C11—C16	−108.35 (14)	C13—C14—C15—C16	1.0 (2)
C2—N1—C11—C12	−119.64 (14)	C13—C14—C15—O15A	178.23 (18)
C5—N1—C2—N3	−0.15 (14)	O15A—C15—C16—C11	−178.53 (15)
C2—N1—C5—C51	179.54 (12)	C14—C15—C16—C11	−1.1 (2)
C11—N1—C5—C4	170.48 (11)	C2—C21—C22—C23	178.55 (12)
C4—N3—C2—N1	0.45 (14)	C26—C21—C22—C23	0.6 (2)
C2—N3—C4—C41	−178.92 (13)	C2—C21—C26—C25	−179.31 (12)
C2—N3—C4—C5	−0.59 (15)	C22—C21—C26—C25	−1.22 (19)
C4—N3—C2—C21	179.14 (11)	C21—C22—C23—C24	0.3 (2)
N1—C2—C21—C26	−151.86 (12)	C22—C23—C24—F4A	179.31 (14)
N3—C2—C21—C22	−148.36 (13)	C22—C23—C24—C25	−0.6 (2)
N3—C2—C21—C26	29.64 (18)	F4A—C24—C25—C26	−179.94 (14)
N1—C2—C21—C22	30.15 (19)	C23—C24—C25—C26	0.0 (2)
N3—C4—C5—N1	0.50 (15)	C24—C25—C26—C21	0.9 (2)

### Hydrogen-bond geometry (Å, °)

**Table:**

Cg1 is the centroid of the N1/C2/N3/C4/C5 ring.

**Table:**

*D*—H···*A*	*D*—H	H···*A*	*D*···*A*	*D*—H···*A*
C23—H23···Cg1^i^	0.93	2.99	3.8714 (16)	159

Symmetry codes: (i) *x*−1/2, −*y*+1/2, *z*−1/2.
